# Supplementary material for: 3D hierarchical graphene matrices enable stable Zn anodes for aqueous Zn batteries
Source: Nat Commun. 2023 Jul 14;14:4205. doi: 10.1038/s41467-023-39947-8 (PMC10349079; doi:10.1038/s41467-023-39947-8)
Supplement: Supplementary file 1 — Supplementary Information [file 41467_2023_39947_MOESM1_ESM.pdf]

Supporting Information

3D Hierarchical Graphene Matrices Enables Stable Zn Anodes for Aqueous Zn Batteries

Yongbiao Mu ‡,[1,2,3] Zheng Li ‡ , [1,2,3] Bu-ke Wu ‡, [1,2,3] Haodong Huang, [1,2,3] Fuhai Wu, [1,2,3] Youqi Chu, [2] Lingfeng Zou, [1,2,3] Ming Yang,[2,4] Jiafeng He, [2,3] Ling Ye, [2] Meisheng Han,[2,3] Tianshou Zhao,[1,2,3] and Lin Zeng * [1,2,3]*

Y. Mu. Z. Li, B. Wu, H. Huang, F. Wu, Y. Chu, L. Zou, M. Yang, J. He, L. Ye, M. Han, Prof. T.S. Zhao, and Prof. L. Zeng

[1]Shenzhen Key Laboratory of Advanced Energy Storage, Southern University of Science and Technology, Shenzhen 518055, China

[2]Department of Mechanical and Energy Engineering, Southern University of Science and Technology, Shenzhen 518055, China

[3]SUSTech Energy Institute for Carbon Neutrality, Southern University of Science and Technology, Shenzhen 518055, China

[4]College of Chemistry and Environmental Engineering, Shenzhen University, Shenzhen 518060, China

‡These authors contributed equally.

E-mail: zhaots@sustech.edu.cn (T.S. Zhao), zengl3@sustech.edu.cn (Lin Zeng)

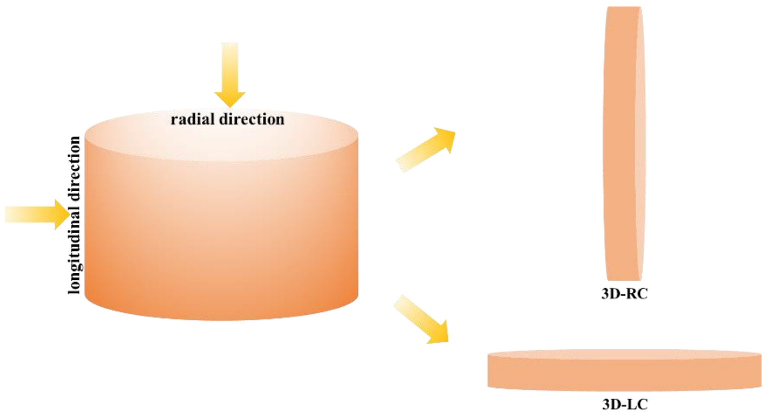

Figure S1. Schematic diagram of the natural basswood processing.

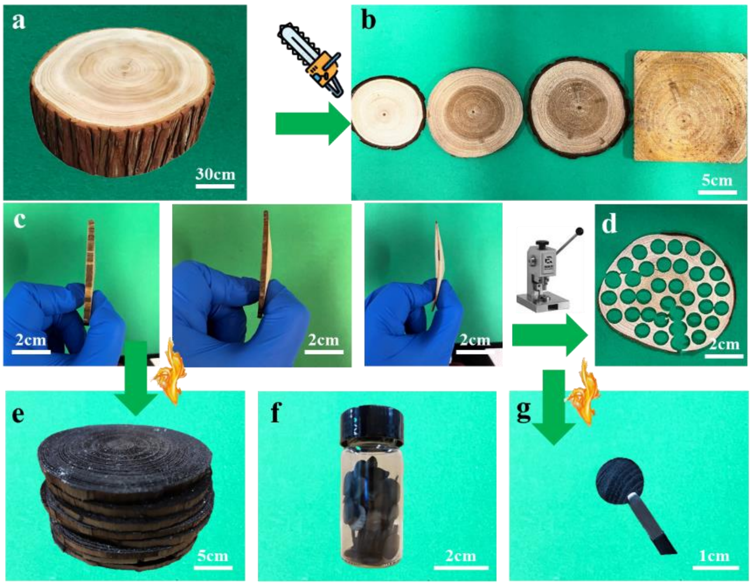

Figure S2. The optical photographs of raw wood and after different treatments including a) wood from the tree; b) different sizes and shapes; c) different thicknesses; d) used in this work and e-g) different size wood-derived multichannel carbon (3D-LC).

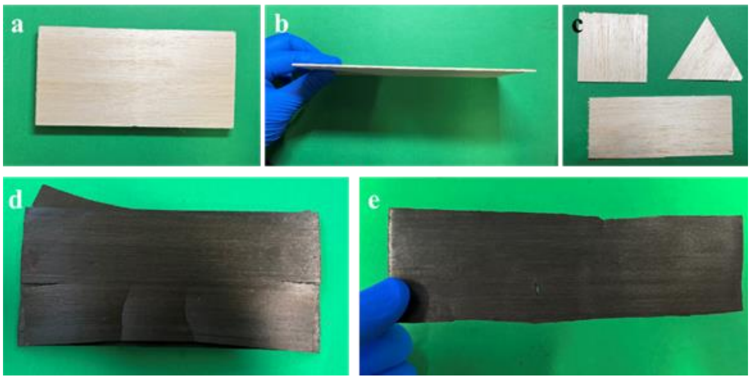

Figure S3. The optical photographs of raw wood and after different treatments including a) wood from the tree; b) cross-sectional image; c) different sizes and shapes; d) pre-oxidation and e) carbonized multichannel carbon (3D-RC).

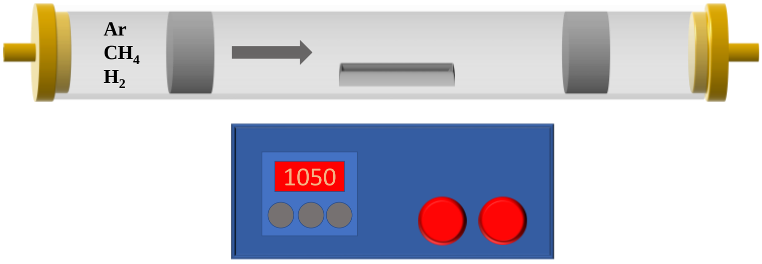

Figure S4. The device of growing GFs and VGs by a thermal-CVD method.

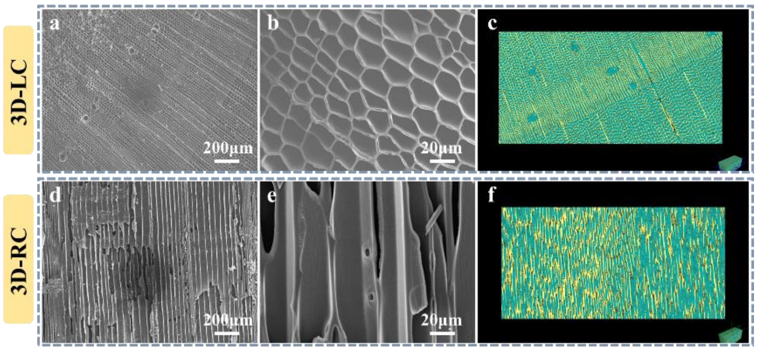

Figure S5. SEM and micro-CT images of 3D-LC and 3D-RC.

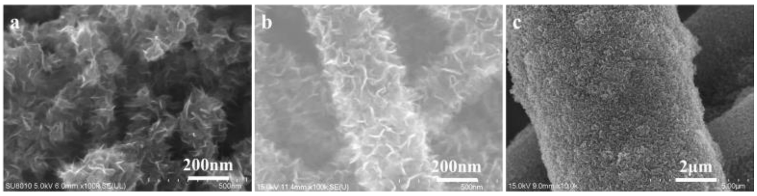

Figure S6. SEM images of vertical graphene grown on a) carbon black, b) carbon nanofibers and c) carbon fibers.

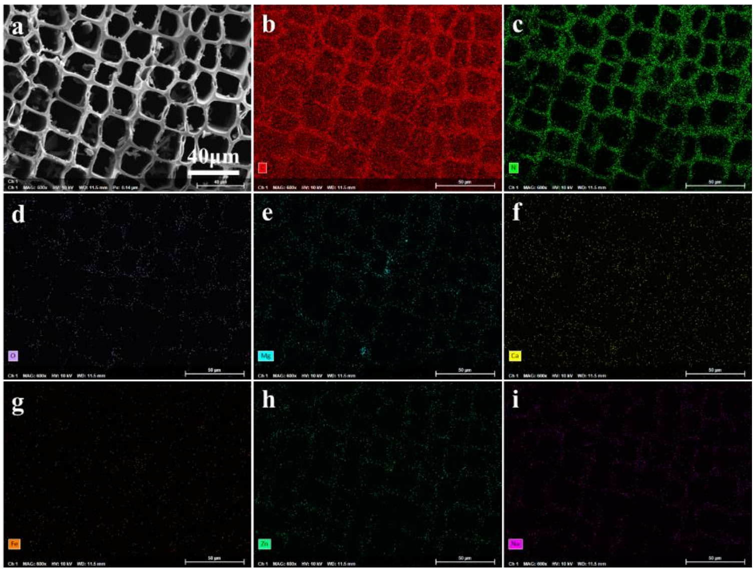

Figure S7. EDX mapping of carbonized wood including a) SEM image, b) C, c) N, d) O, e) Mg, f) Ca, g) Fe, h) Zn, i) Na elements. (The scale bar is 40 μm)

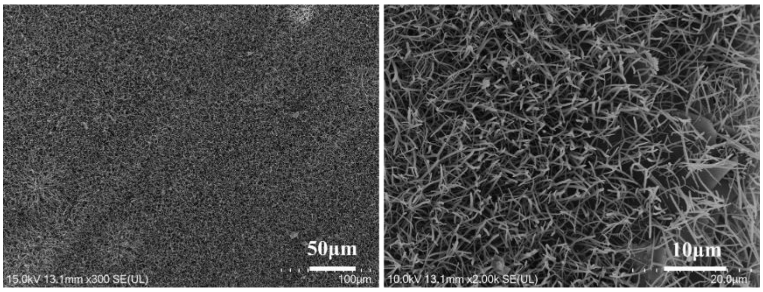

Figure S8. SEM image of 3D-RFGC in the presence of a Fe-based catalyst.

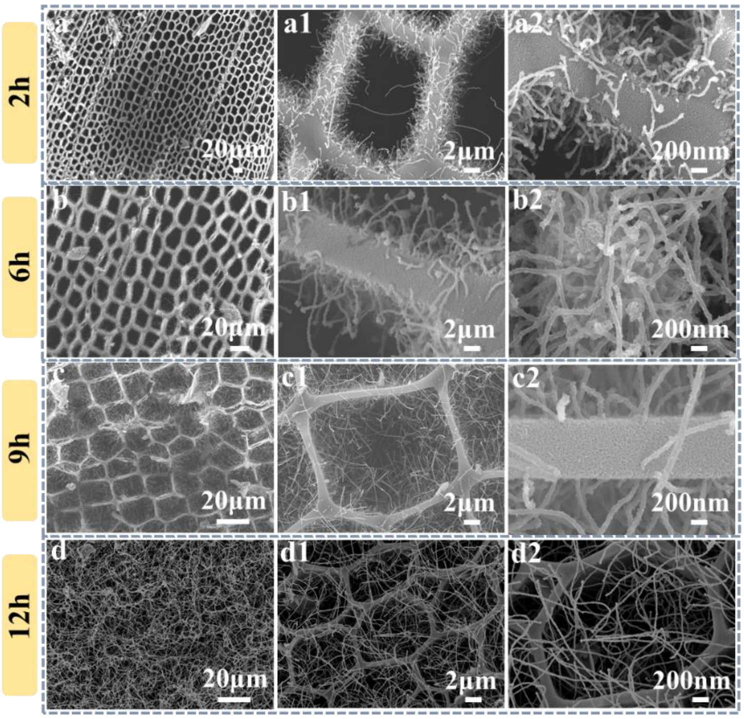

Figure S9. SEM images of 3D-LFGC matrices for longitudinal direction when extended the growth time a) 2 h; b) 6 h; c) 9 h and d)12 h.

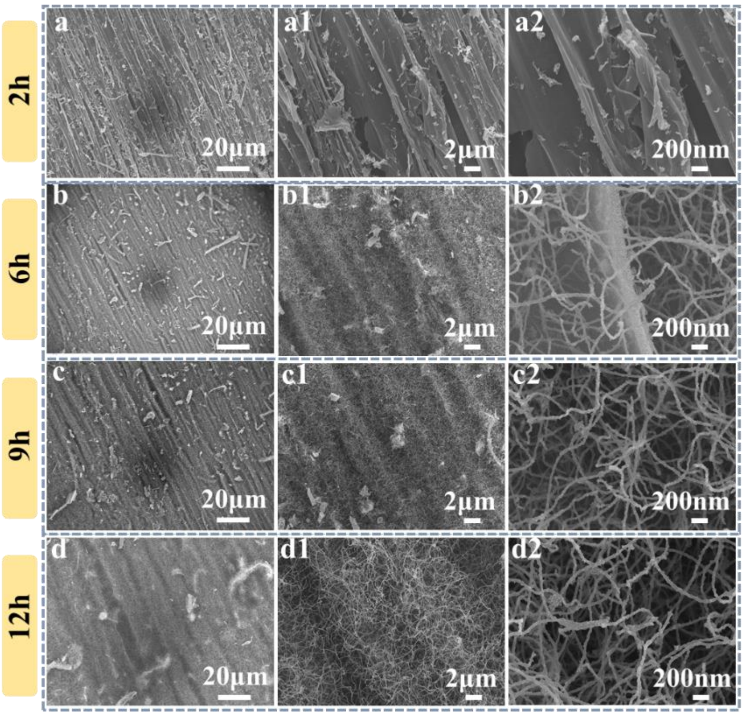

Figure S10. SEM images of 3D-RFGC matrices for longitudinal direction when extended the growth time a) 2 h; b) 6 h; c) 9 h and d) 12 h.

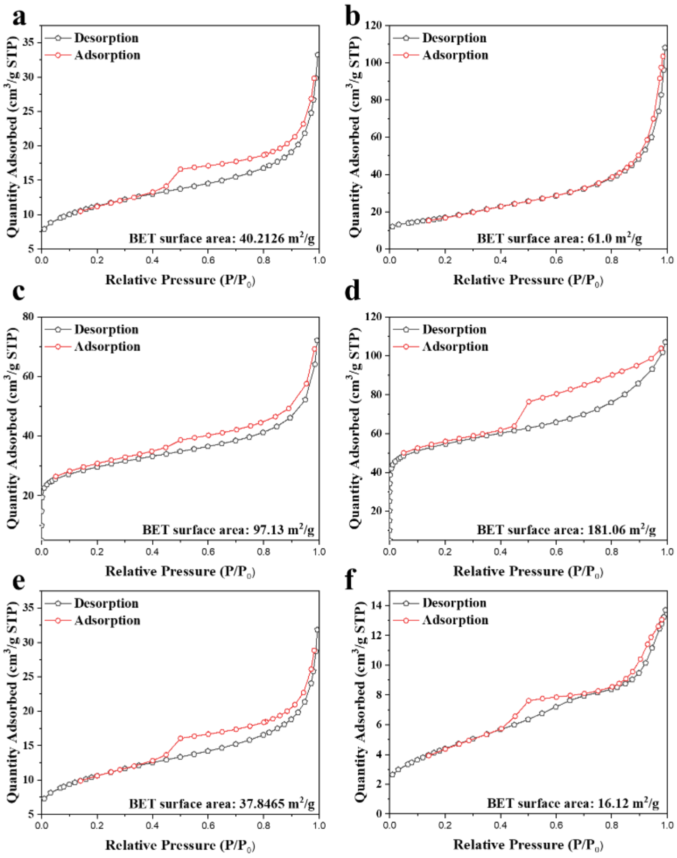

Figure S11. N_2 adsorption–desorption isotherms of a) 3D-LC, b) 3D-RC, c) 3D-LFGC, d) 3D-RFGC, e) 3D-LFGC@Zn, f) 3D-RFGC@Zn.

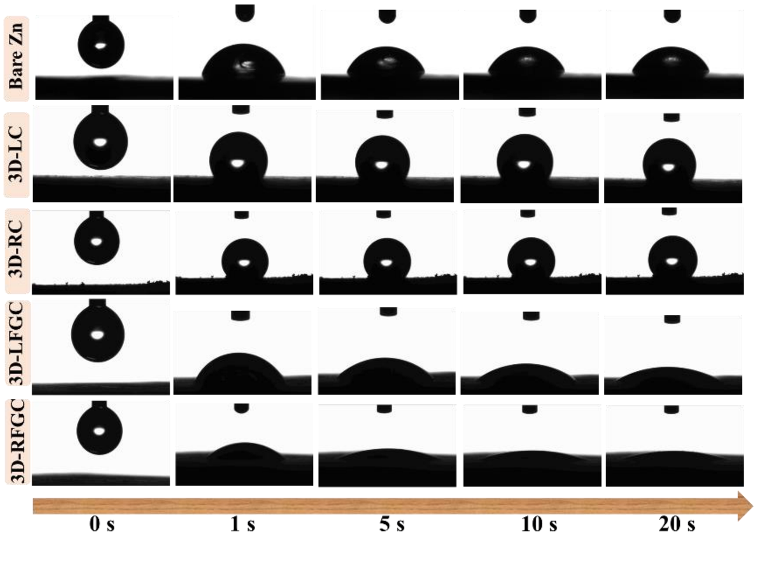

Figure S12. The optical images of contact angles on various current collectors in this work.

Table S1. The contact angles of bare Zn and various matrices used in this work.

Samples	1 s	5 s	10 s	20 s
Bare Zn	51.96	43.63	40.35	40.35
3D-LC	134.10	133.50	133.50	133.50
3D-RC	121.76	120.30	120.10	120.10
3D-LFGC	54.31	39.57	28.28	22.92
3D-RFGC	29.51	14.61	9.68	7.92

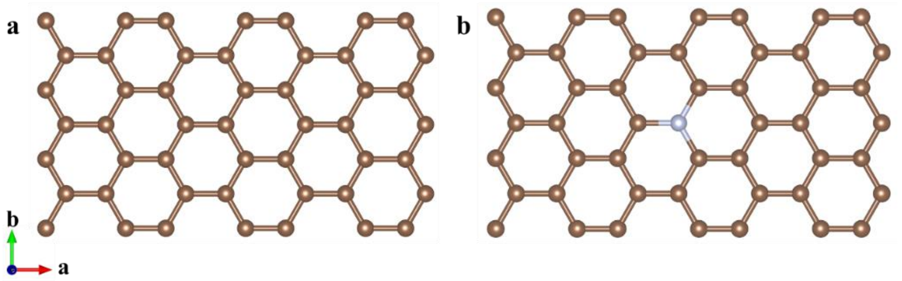

Figure S13. Configurations of graphene (a) and nitrogen-doped graphene (b).

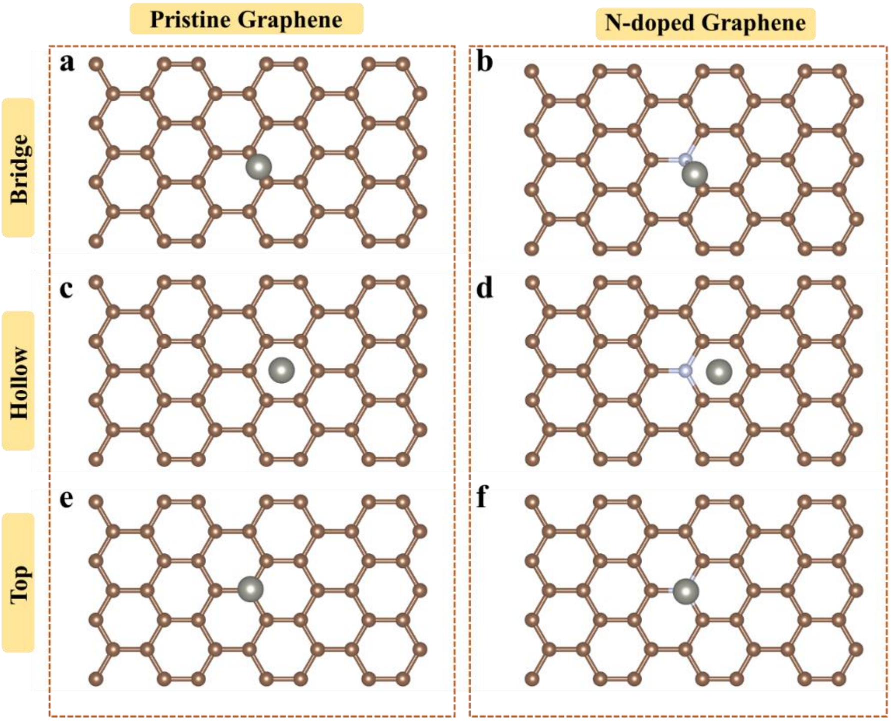

Figure S14. Configurations of zinc adsorption on different sites of graphene/nitrogen-doped graphene.

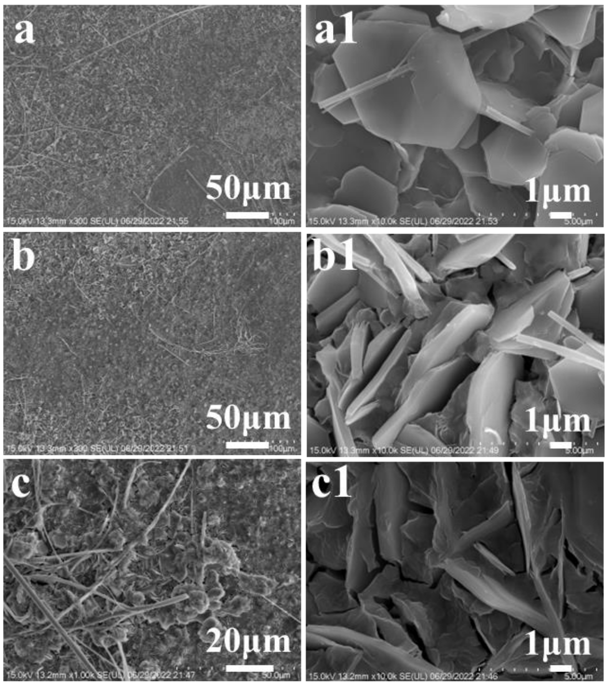

Figure S15. SEM images of Zn metal deposited on the 2D Cu foil for a) 1 mAh cm^{-2}; b) 2 mAh cm^{-2}; c) 5 mAh cm^{-2}.

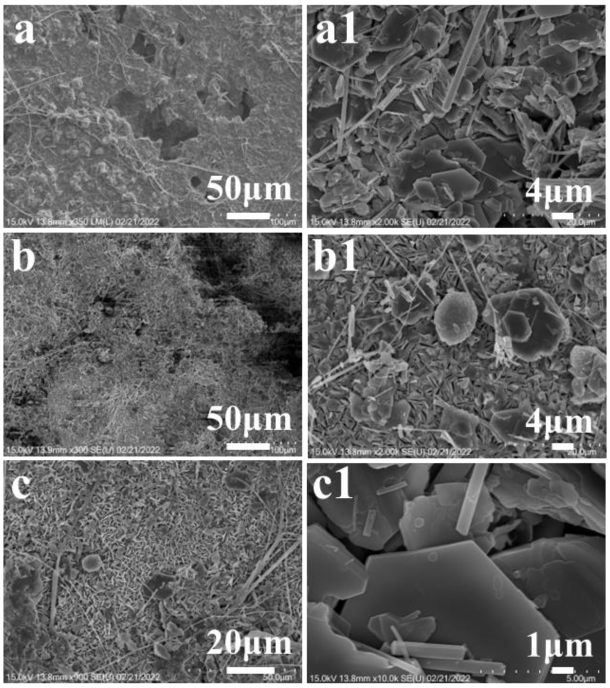

Figure S16. SEM images of Zn metal deposited on the Zn foil for a) 1 mAh cm^{-2}; b) 2 mAh cm^{-2}; c) 5 mAh cm^{-2}.

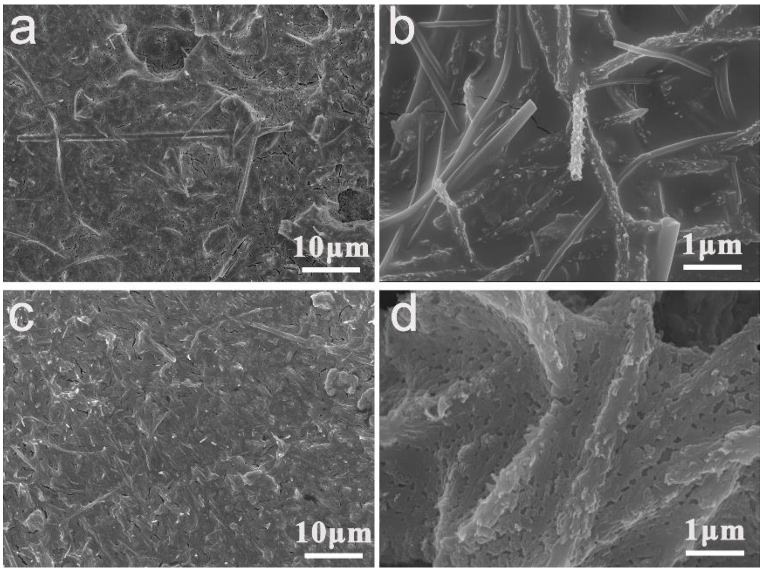

Figure S17. SEM images of Zn metal deposited on the a, b) 3D-LFGC; c, d) 3D-RFGC with homogeneous and dense surface morphologies.

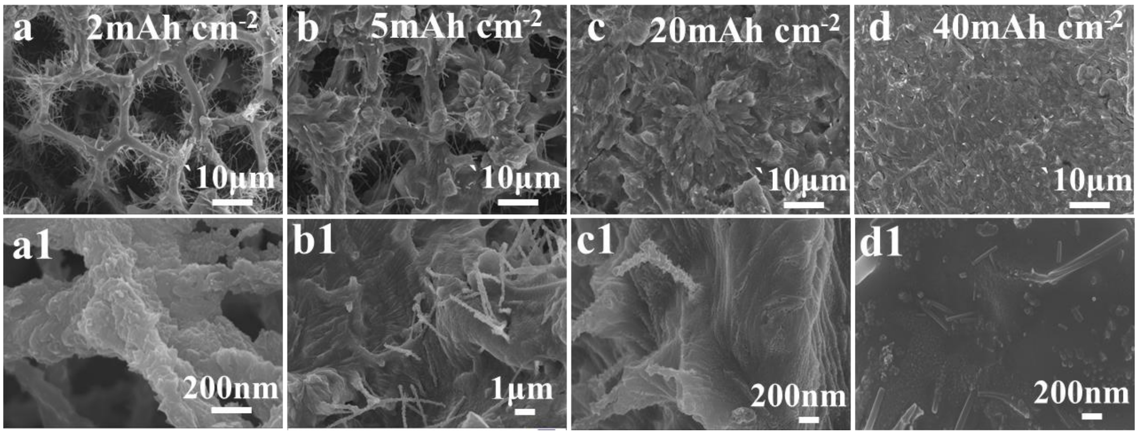

Figure S18. SEM images of Zn metal deposited on the 3D-LFGC matrices for a, a1) 2 mAh cm^{-2}; b, b1) 5 mAh cm^{-2}; c, c1) 20 mAh cm^{-2} and d, d1) 40 mAh cm^{-2}.

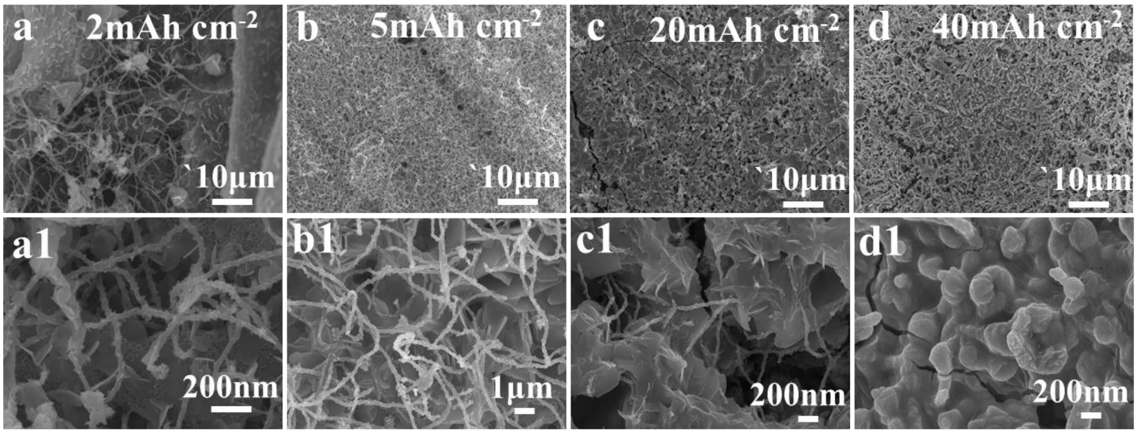

Figure S19. SEM images of Zn metal deposited on the 3D-RFGC matrices for a, a1) 2 mAh cm^{-2}; b, b1) 5 mAh cm^{-2}; c, c1) 20 mAh cm^{-2} and d, d1) 40 mAh cm^{-2}.

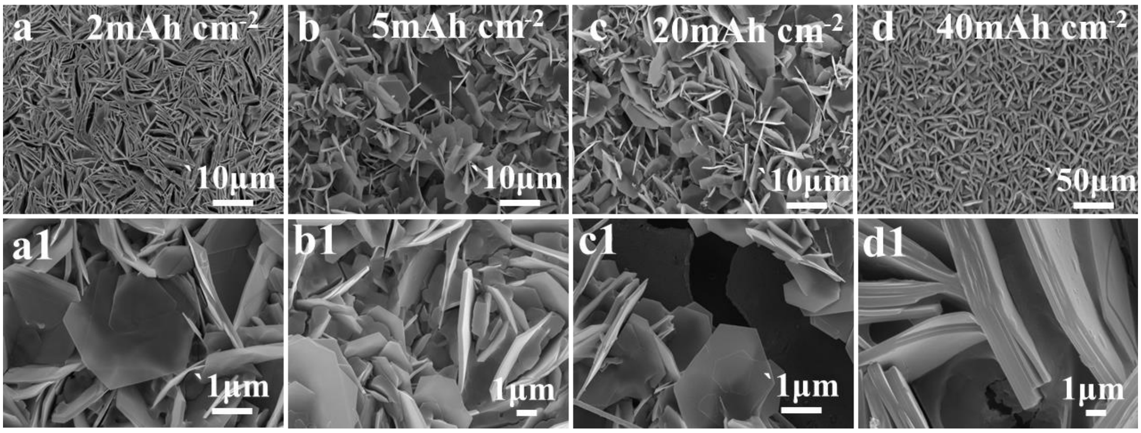

Figure S20. SEM images of Zn metal deposited on the Zn foil for a, a1) 2 mAh cm^{-2}; b, b1) 5 mAh cm^{-2}; c, c1) 20 mAh cm^{-2} and d, d1) 40 mAh cm^{-2}.

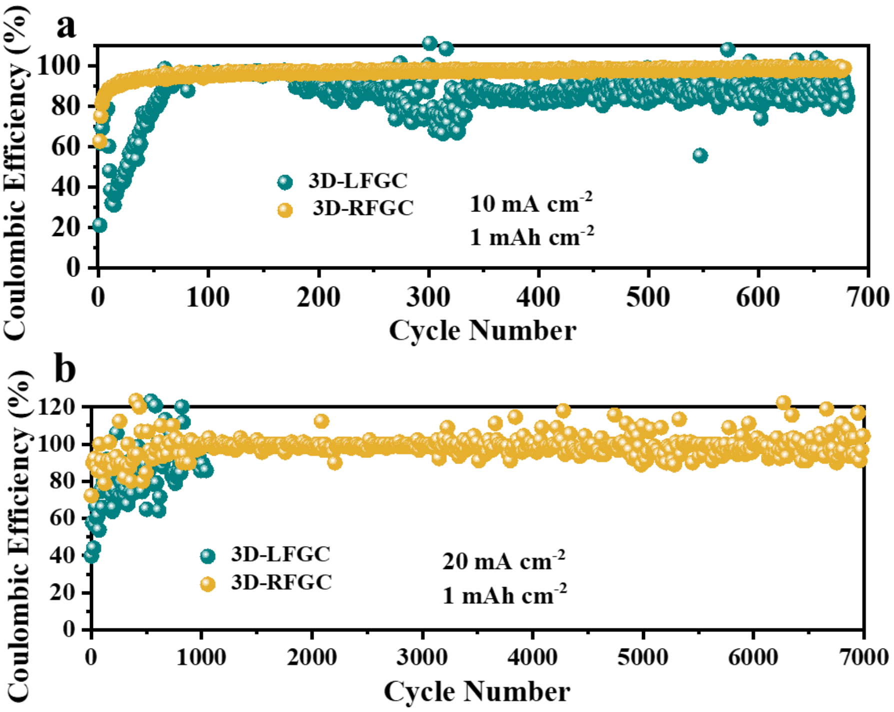

Figure S21. CE plots of different matrices at a current density of a) 10 mA cm^{-2} and b) 20 mA cm^{-2} with a capacity of 1 mAh cm^{-2}.

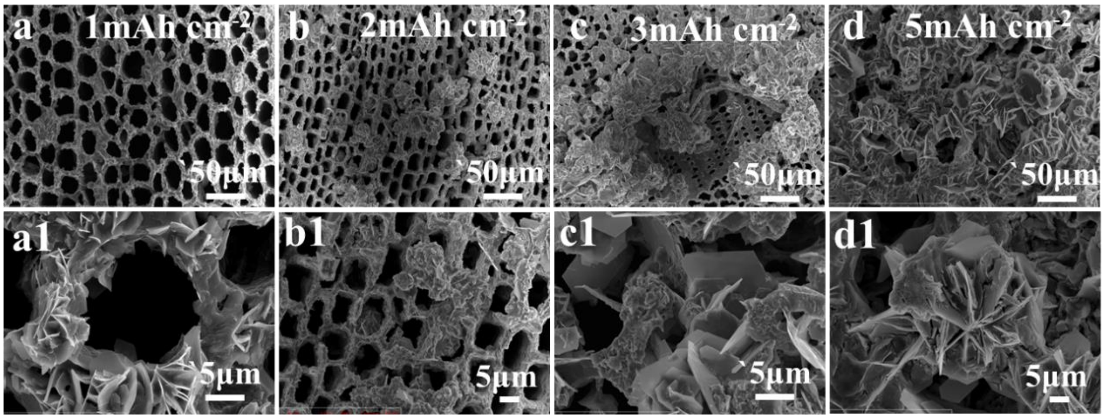

Figure S22. SEM images of Zn metal deposited on the 3D-LC without GFs and VGs for a, a1) 1 mAh cm^{-2}; b, b1) 2 mAh cm^{-2}; c, c1) 3 mAh cm^{-2} and d, d1) 5 mAh cm^{-2}.

Table S2. Comparison of CE of our 3D-RFGC@Zn anode and reported materials for ZMAs. (C_d: current density; C: capacity; L: lifespan; CE: coulombic efficiency)

Samples	C_d (mA cm^{-2})	C (mAh cm^{-2})	L (cycles)	CE (%)	Reference
CuZIF-L@TM	1	1	300	97.60	[6]
TZNC	5	1	1000	98.60	[7]
MGA@Zn	10	1	600	99.67	[8]
CnC HS	4	1	200	95.00	[9]
3D Cu@Zn	1	2	66	99.53	[10]
3D Ti/Zn	10	5	200	93.69	[11]
3DGs@Zn	80	0.5	400	95.68	[12]
Sn-PCF	10	1	1000	99.80	[13]
NSH	5	1	200	99.50	[14]
Zn/CNT	5	2	30	97.90	[15]
Cu-Ps/EG	1	1	300	90.00	[16]
AgNWA@Zn	40	10	200	99.30	[17]
3D-Topo-Zn	1	1	1300	99.50	[18]
**3D-RFGC@Zn**	**1**	**0.5**	**400**	**99.52**	**This work**
**3D-RFGC@Zn**	**10**	**0.5**	**1000**	**99.40**	**This work**
**3D-RFGC@Zn**	**40**	**0.5**	**3000**	**99.38**	**This work**
**3D-RFGC@Zn**	**40**	**8**	**50**	**98.60**	**This work**
**3D-RFGC@Zn**	**40**	**40**	**20**	**97.50**	**This work**
**3D-RFGC@Zn**	**80**	**0.5**	**5000**	**99.20**	**This work**
**3D-RFGC@Zn**	**120**	**0.5**	**2000**	**98.90**	**This work**
**3D-RFGC@Zn**	**120**	**5**	**150**	**98.80**	**This work**

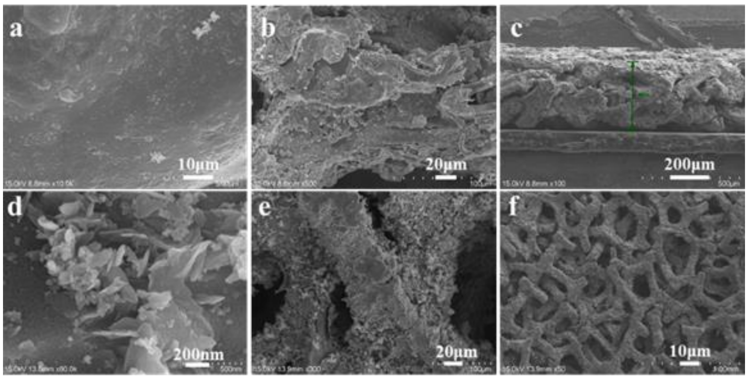

Figure S23. SEM images of commercial foam Zn from a-c) cross-sectional view and d-f) top-view.

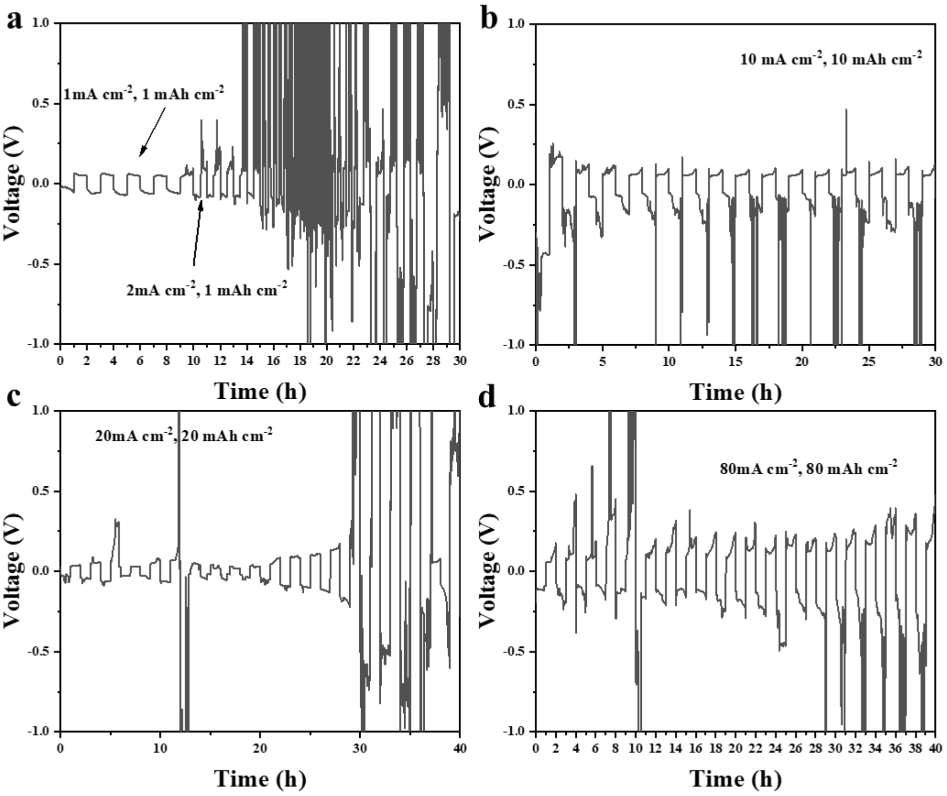

Figure S24. Symmetrical cells of 3D Zn foam operating at a) 1 mA cm^{-2} and 1 mAh cm^{-2}; b) 10 mA cm^{-2} and 10 mAh cm^{-2}; c) 20 mA cm^{-2} and 20 mAh cm^{-2}; d) 80 mA cm^{-2} and 80 mAh cm^{-2}.

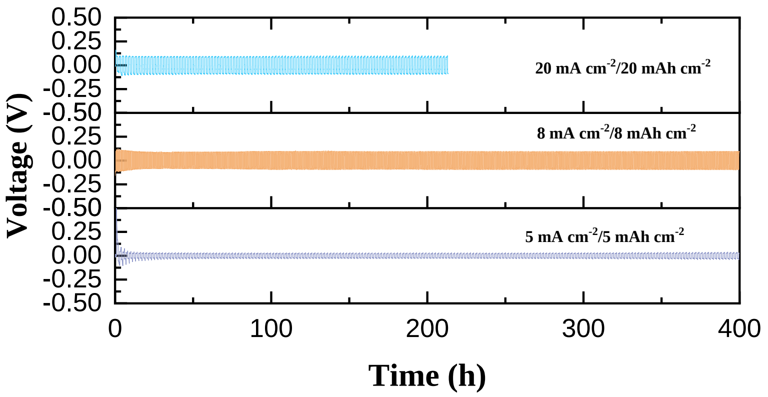

Figure S25. Galvanostatic cycling of 3D-RFGC@Zn symmetrical cells at a) 5 mA cm^{-2} and 5 mAh cm^{-2}, b) 8 mA cm^{-2} and 8 mAh cm^{-2}, c) 20 mA cm^{-2} and 20 mAh cm^{-2}.

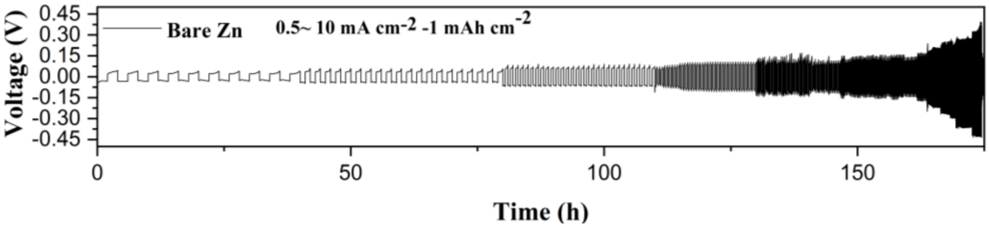

Figure S26. Rate performance of Zn/Zn symmetrical cells.

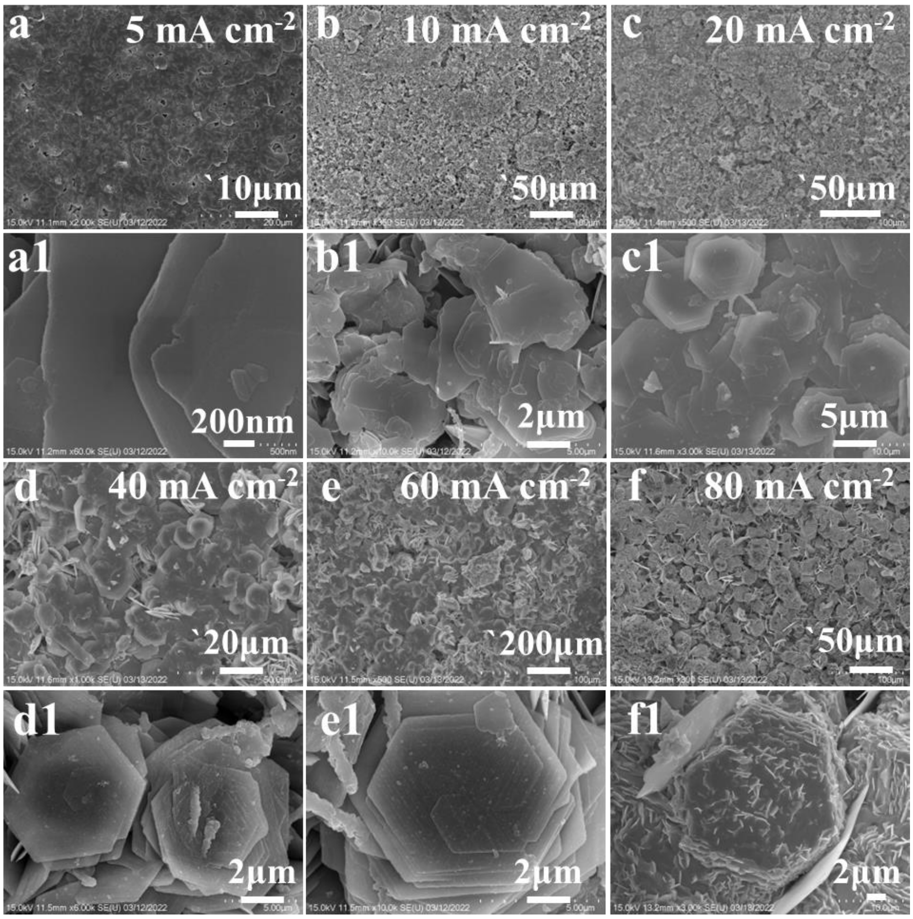

Figure S27. SEM images of Zn metal deposited on the 3D-RFGC matrices under different current densities of a, a1) 5 mA cm^{-2}, b, b1) 10 mA cm^{-2}, c, c1) 20 mA cm^{-2}, d, d1) 40 mA cm^{-2}, e, e1) 60 mA cm^{-2}, and f, f1) 80 mA cm^{-2}.

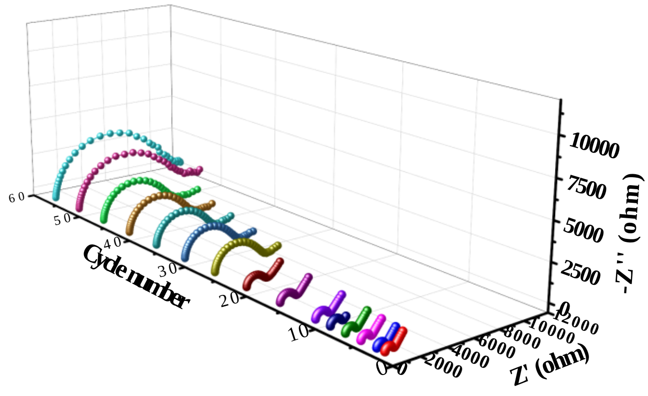

Figure S28. In situ EIS curves of Zn/Zn symmetric cells during continuous Zn plating process.

Table S3. The values of R_{ct} of in-situ EIS curves of 3D-RFGC@Zn/3D-RFGC@Zn and

bare Zn/Zn symmetric cells during continuous Zn plating process. (C_n-cycle number (n); R_{ct}-charge transfer resistance)

C_n (n)	R_{ct} (Ω)	C_n (n)	R_{ct} (Ω)
3D-RFGC@Zn/3D-RFGC@Zn		Zn/Zn	
1	182.54	1	622.32
2	192.64	2	658.97
4	190.46	4	720.89
6	214.47	6	782.64
8	190.50	8	907.09
10	204.93	10	1008.60
15	200.53	15	1192.80
20	203.95	20	1524.30
30	214.47	25	3914.90
40	218.04	30	4194.30
50	228.32	35	4862.30
55	231.86	40	5333.50
70	247.92	45	6165.30
/	/	50	8712.50
/	/	55	10210.00

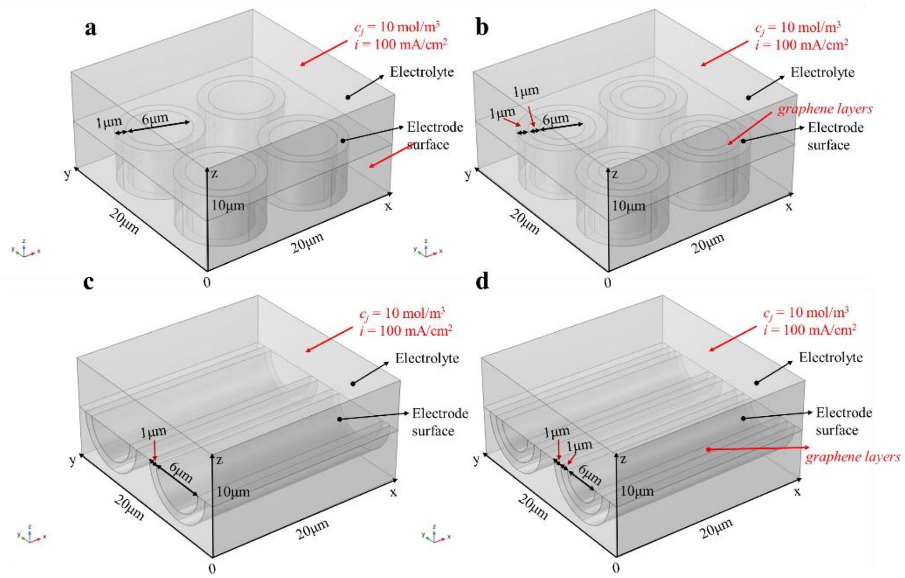

Figure S29. The geometrical structures and boundaries condition for (a) 3D-LC; (b) 3D-LFGC; (c) 3D-RC and (d) 3D-RFGC.

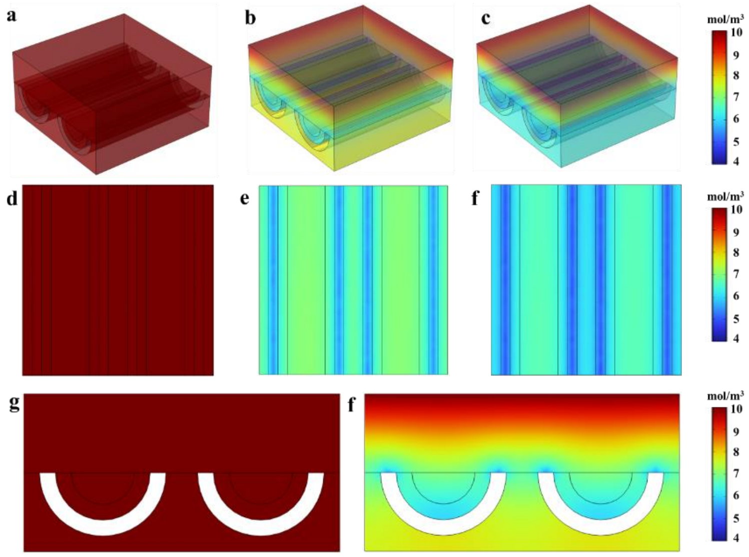

Figure S30. Simulation results of the Zn^{2+} ions concentration distribution at different stages for 3D-RFGC. a, d, g) 3D model, top-view and vertical-view at 0 s; b, e, h) 3D model, top-view and vertical-view at 60 s; c, f) 3D model, top-view at 120 s.

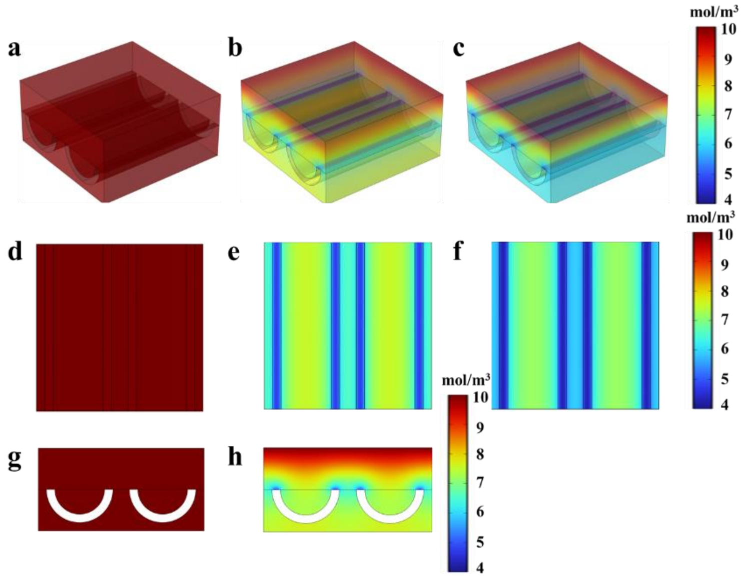

Figure S31. Simulation results of the Zn^{2+} ions concentration distribution at different stages for 3D-RC. a, d, g) 3D model, top-view and vetical-view at 0 s; b, e, h) 3D model, top-view and vetical-view at 60 s; c, f) 3D model, top-view at 120 s.

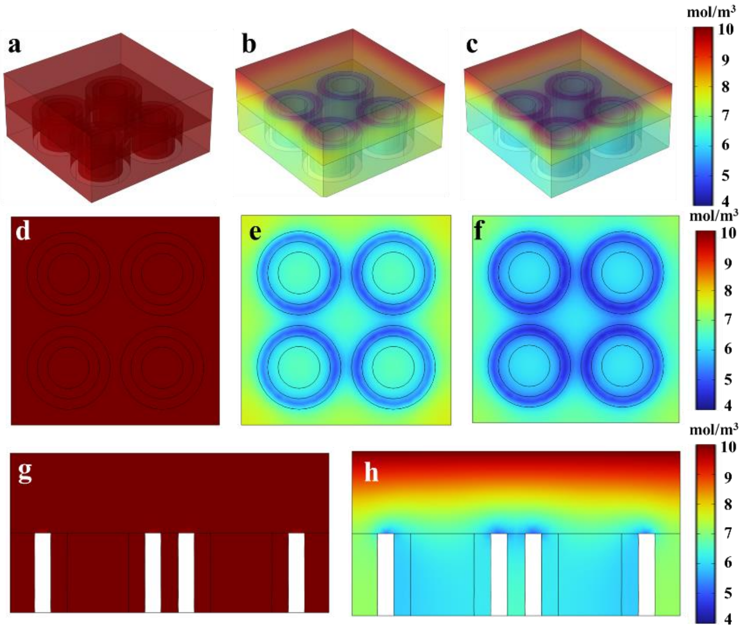

Figure S32. Simulation results of the Zn^{2+} ions concentration distribution at different stages for 3D-LFGC. a, d, g) 3D model, top-view and vetical-view at 0 s; b, e, h) 3D model, top-view and vetical-view at 60 s; c, f) 3D model, top-view at 120 s.

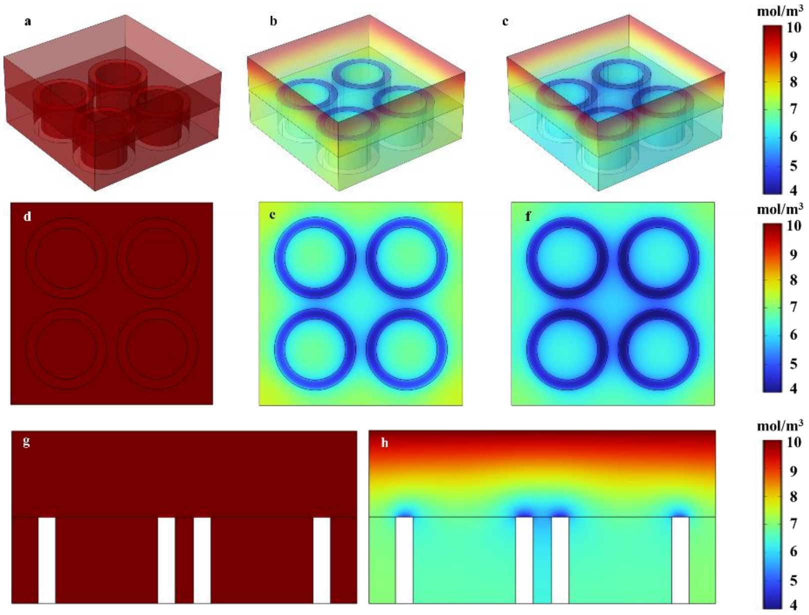

Figure S33. Simulation results of the Zn^{2+} ions concentration distribution at different stages for 3D-LC. a, d, g) 3D model, top-view and vetical-view at 0 s; b, e, h) 3D model, top-view and vetical-view at 60 s; c, f) 3D model, top-view at 120 s.

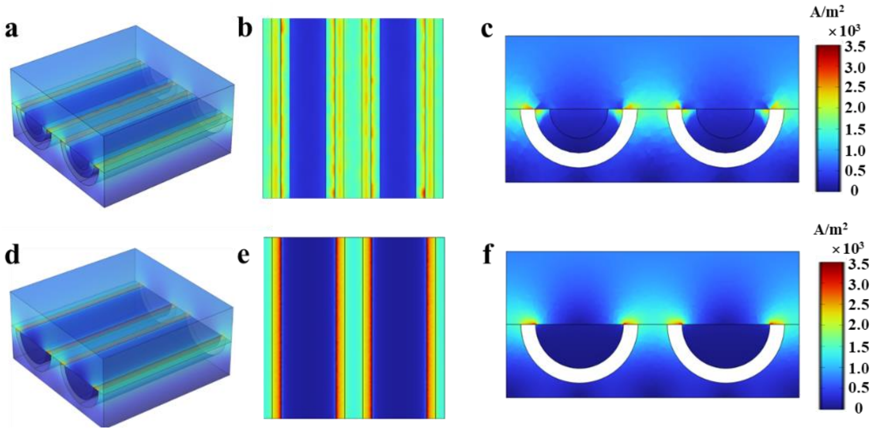

Figure S34. Simulation results of the local current density distribution for 3D-RFGC and 3D-RC. a, d) 3D model, b, e) top-view and c, f) vertical-views.

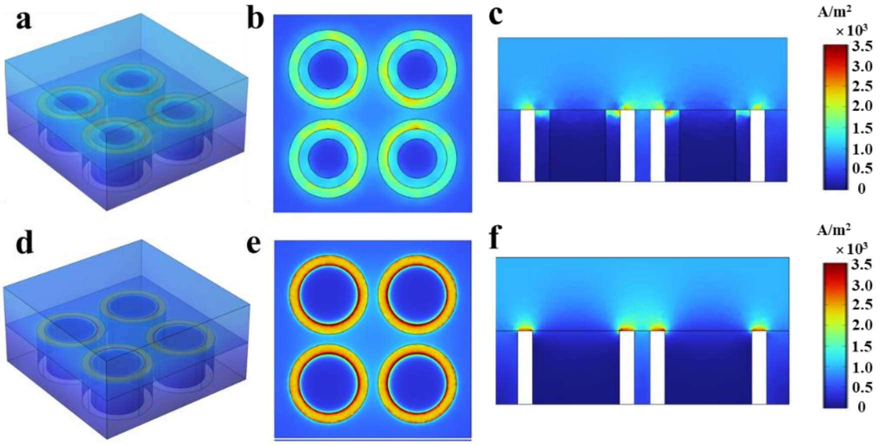

Figure S35. Simulation results of the local current density distribution for 3D-LFGC and 3D-LC. a, d) 3D model, b, e) top-view and c, f) vetical-views.

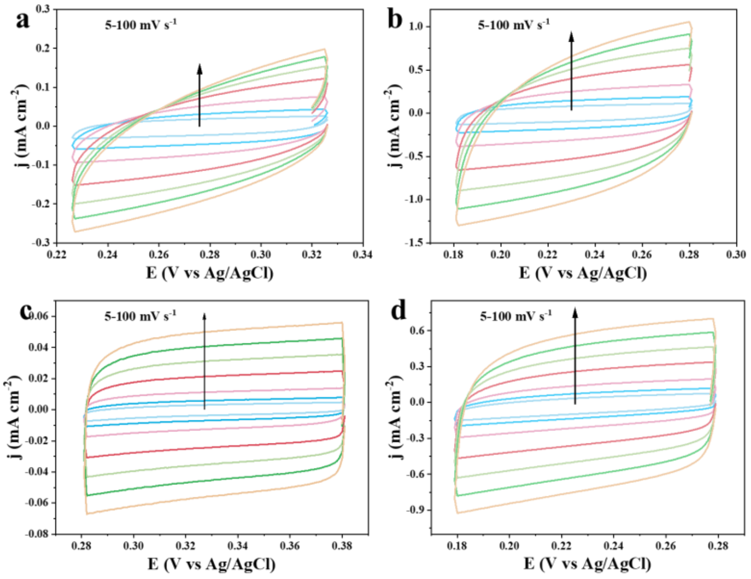

Figure S36. Electrochemically active surface area (ECSA) measurements of a) 3D-LC, b) 3D-RC, c) 3D-LFGC and d) 3D-RFGC. The CV curves of a) 3D-LC, b) 3D-RC, c) 3D-LFGC and d) 3D-RFGC electrodes, respectively, at various scan rates ranging from 5 to 100 mV s^{-1} in 2 M $ZnSO_4$.

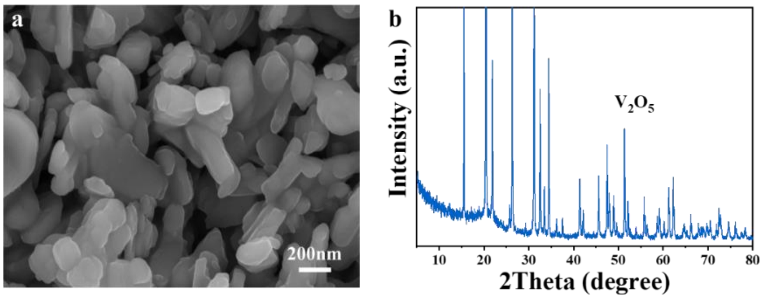

Figure S37. a) SEM image and b) XRD data of commercial V_2O_5 cathode material.

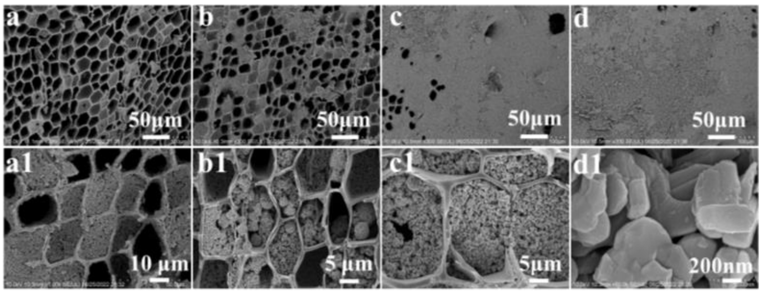

Figure S38. SEM images of V_2O_5@3D-LC composite cathode with mass loading of a, a1) 4 mg cm^{-2}, b, b1) 12.5 mg cm^{-2}, c, c1) 22.6 mg cm^{-2}, and d, d1) 35 mg cm^{-2}.

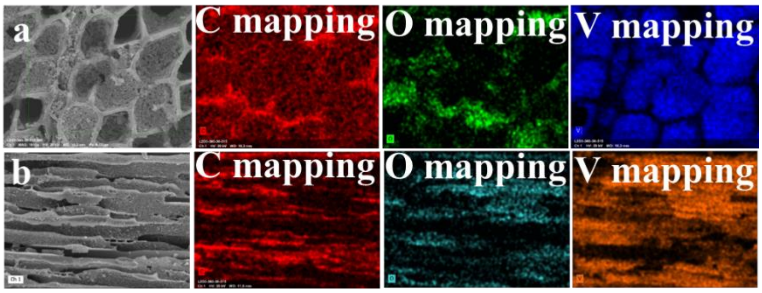

Figure S39. EDS mapping of V_2O_5@3D-LC cathode from a) top-view and b) cross-view.

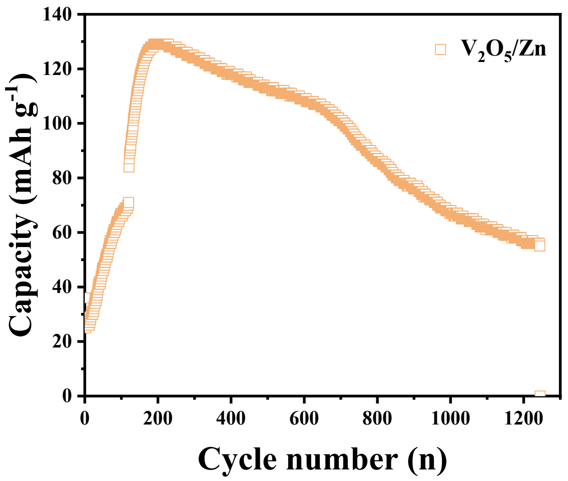

Figure S40. The cycle performance of the V_2O_5//Zn full cell at 40.0 mA cm^{-2}.

Table S4. The values of R_{ct} in-situ EIS spectra of V_2O_5@3D-LC/3D-RFGC@Zn full cells electrode before cycling and cycling at different charge/discharge stages.

C_n (n)	R_{ct} (Ω)	C_n (n)	R_{ct} (Ω)
1	200.610	16	27.941
2	196.980	17	26.072
3	190.250	18	24.614
4	172.950	19	23.292
5	174.910	20	22.175
6	161.880	21	21.284
7	133.950	22	20.482
8	94.4640	23	18.954
9	70.348	24	18.089
10	56.164	25	17.613
11	46.916	26	17.365
12	40.758	27	16.910
13	36.449	/	/
14	33.052	/	/

15	30.100	/	/

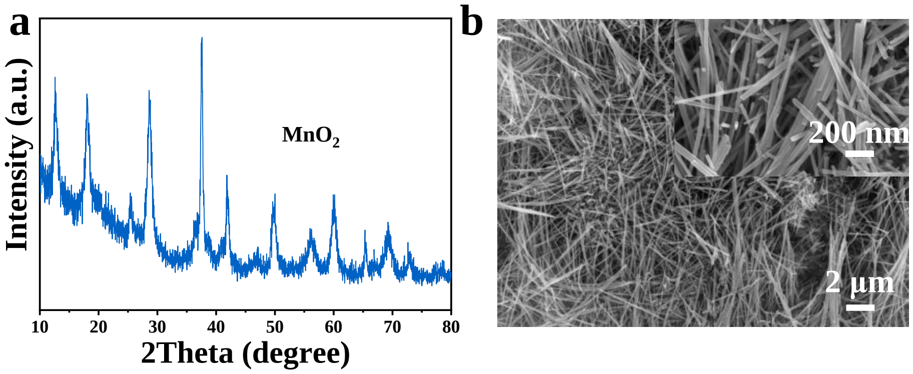

Figure S41. a) XRD and b) SEM image of commercial MnO_2 cathode material.

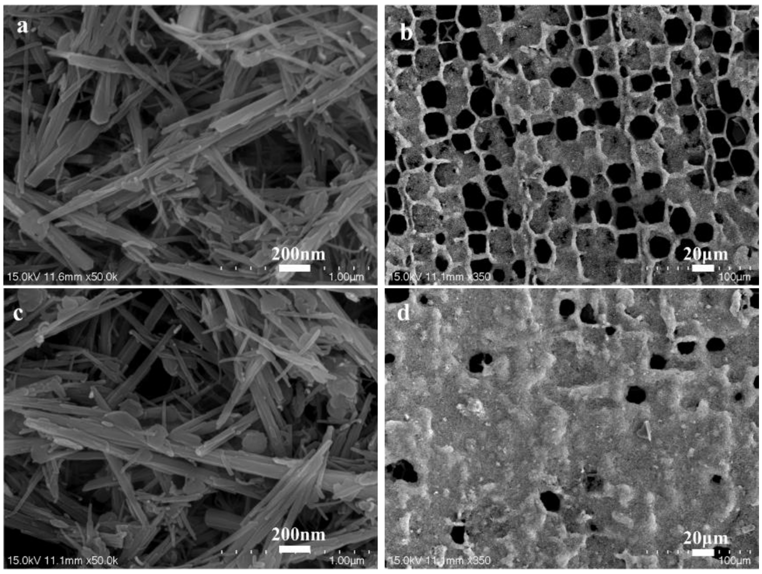

Figure S42. SEM images of MnO_2@3D- LC at a, b) low mass loading and c, d) high mass loading.

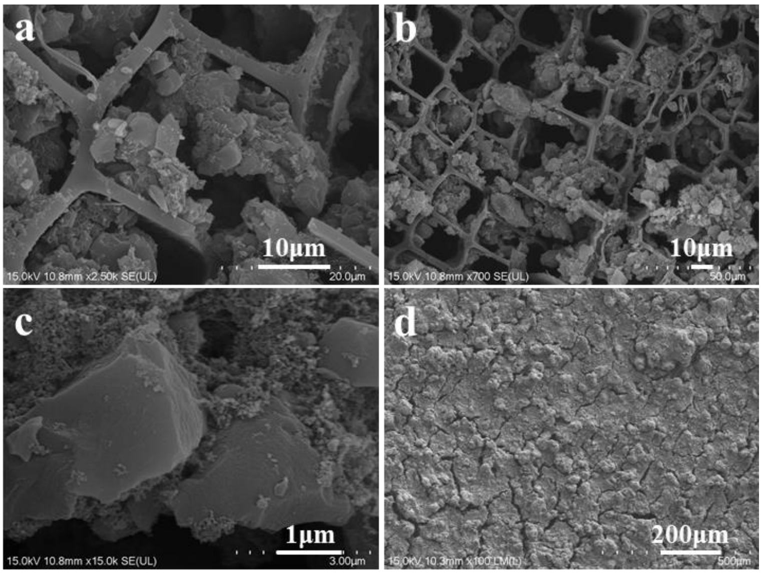

Figure S43. SEM images of 3D- LC@AC at a, b) low mass loading and c, d) high mass loading.

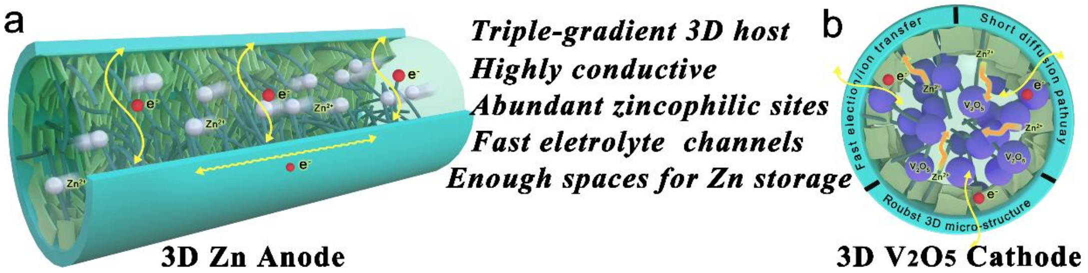

Figure S44. Functional description of our 3D host for Zn anode and cathodes (V_2O_5 or α-MnO_2 and AC cathodes).

Table S5 Comparison of electrochemical performances of our 3D-RFGC@Zn anode and reported materials for ZMAs. (C_d: current density; C: capacity; L:lifespan, V_h: voltage hysteresis; DOD: depth-of-discharge)

Samples	C_d (mA cm^{-2})	C (mAh cm^{-2})	L (h)	V_h (mV)	DOD (%)	Reference
CuZIF-L@TM	6	7	650	80	50	[6]
TZNC	1	1	450	12	50	[7]
3D Ni-Zn	5	2	300	/	/	[19]
MXene/Graphene Aerogel	10	1	1050	64	60	[8]
CnC HS	4	1	116	40	/	[9]
3D Zn	10	1	190	45	/	[10]
3D Ti−TiO_2	10	0.5	500	/	/	[11]
3DGT@Zn	2	1	1100	/	47.1	[12]
3D SnPCF	10	5	500	47	/	[13]
Zn/CNT	5	2.5	110	68	28	[15]
Cu-Ps/EG	10	10	3000	71.4	/	[16]
AgNWA@Zn//C-MOF	40	10	90	/	/	[17]
3D-Topo-Zn	2	2	1160	/	11.4	[18]
3D-ZGC	20	1	150	65	/	[20]
3DP-ZA	4	2	180	/	/	[21]
Zn@PCH	10	1	110		/	[14]
SS-V_2O_3@C/Zn	20	20	5000	/	80	[22]
3D-Zn	5	5	160	/	/	[23]
Ti_3C_2Tx MXene/ZnS	10	1	180	/	/	[24]
N-VG@CC	1	1	70		/	[25]
FPCH-ZI/Zn	10	5	650	43.5	51	[26]
**3D-RFGC@Zn**	**1**	**1**	**7300**	**15**	**20**	**This work**
**3D-RFGC@Zn**	**20**	**20**	**2000**	**32**	**50**	**This work**
**3D-RFGC@Zn**	**40**	**40**	**2200**	**26**	**100**	**This work**
**3D-RFGC@Zn**	**80**	**80**	**2600**	**31**	**100**	**This work**

Supplementary references

1. Mu Y*, et al.* Nitrogen, Oxygen-Codoped Vertical Graphene Arrays Coated 3D Flexible Carbon Nanofibers with High Silicon Content as an Ultrastable Anode for Superior Lithium Storage. *Adv Sci (Weinh)* **9**, e2104685 (2022).
2. Ji X*, et al.* High yield production of 3D graphene powders by thermal chemical vapor deposition and application as highly efficient conductive additive of lithium ion battery electrodes. *Carbon*, (2021).
3. Ji X*, et al.* Graphene/MoS_2/FeCoNi$(OH)_x$ and Graphene/MoS_2/$FeCoNiP_x$ multilayer-stacked vertical nanosheets on carbon fibers for highly efficient overall water splitting. *Nature Communications*, (2021).
4. Tian ZQ*, et al.* A Highly Order-Structured Membrane Electrode Assembly with Vertically Aligned Carbon Nanotubes for Ultra-Low Pt Loading PEM Fuel Cells. *Advanced Energy Materials* **1**, 1205-1214 (2011).
5. Wu B*, et al.* Graphene Scroll-Coated alpha-MnO_2 Nanowires as High-Performance Cathode Materials for Aqueous Zn-Ion Battery. *Small* **14**, e1703850 (2018).
6. Tao Y*, et al.* Atomically Dispersed Cu in Zeolitic Imidazolate Framework Nanoflake Array for Dendrite-Free Zn Metal Anode. *Small* **18**, e2203231 (2022).
7. Sun PX*, et al.* Formation of Super-Assembled TiO_x/Zn/N-Doped Carbon Inverse Opal Towards Dendrite-Free Zn Anodes. *Angew Chem Int Ed Engl* **61**, e202115649 (2022).
8. Zhou J*, et al.* Encapsulation of Metallic Zn in a Hybrid MXene/Graphene Aerogel as a Stable Zn Anode for Foldable Zn-Ion Batteries. *Adv Mater* **34**, e2106897 (2022).
9. Xie F*, et al.* Mechanism for Zincophilic Sites on Zinc-Metal Anode Hosts in Aqueous Batteries. *Advanced Energy Materials* **11**, (2021).
10. Kang Z*, et al.* 3D Porous Copper Skeleton Supported Zinc Anode toward High Capacity and Long Cycle Life Zinc Ion Batteries. *ACS Sustainable Chemistry & Engineering* **7**, 3364-3371 (2019).
11. An Y, Tian Y, Xiong S, Feng J, Qian Y. Scalable and Controllable Synthesis of Interface-Engineered Nanoporous Host for Dendrite-Free and High Rate Zinc Metal Batteries. *ACS Nano* **15**, 11828-11842 (2021).
12. Buke W*, et al.* High Zinc Utilization Aqueous Zinc Ion Batteries Enabled by 3D Printed Graphene Arrays. *Energy Storage Materials*, (2022).
13. Yang J-L, Yang P, Yan W, Zhao J-W, Fan HJ. 3D zincophilic micro-scaffold enables stable Zn deposition. *Energy Storage Materials* **51**, 259-265 (2022).
14. Jian Q, Guo Z, Zhang L, Wu M, Zhao T. A hierarchical porous tin host for dendrite-free, highly reversible zinc anodes. *Chemical Engineering Journal* **425**, (2021).
15. Zeng Y*, et al.* Dendrite-Free Zinc Deposition Induced by Multifunctional CNT Frameworks for Stable Flexible Zn-Ion Batteries. *Adv Mater* **31**, e1903675 (2019).
16. Chen G*, et al.* Reversible and homogenous zinc deposition enabled by in-situ grown Cu particles on expanded graphite for dendrite-free and flexible zinc metal anodes. *Energy Storage Materials* **50**, 589-597 (2022).
17. Ling W*, et al.* An ultrahigh rate dendrite-free Zn metal deposition/striping enabled by silver nanowire aerogel with optimal atomic affinity with Zn. *Energy Storage Materials* **51**, 453-464 (2022).

18. Yan M*, et al.* Constructing Three-Dimensional Topological Zn Deposition for Long-Life Aqueous Zn-Ion Batteries. *ACS Appl Mater Interfaces* **14**, 51010-51017 (2022).
19. Zhang G, Zhang X, Liu H, Li J, Chen Y, Duan H. 3D-Printed Multi-Channel Metal Lattices Enabling Localized Electric-Field Redistribution for Dendrite-Free Aqueous Zn Ion Batteries. *Advanced Energy Materials* **11**, (2021).
20. Xue P*, et al.* A MOF-Derivative Decorated Hierarchical Porous Host Enabling Ultrahigh Rates and Superior Long-Term Cycling of Dendrite-Free Zn Metal Anodes. *Adv Mater* **34**, e2110047 (2022).
21. Zeng L*, et al.* Direct 3D printing of stress-released Zn powder anodes toward flexible dendrite-free Zn batteries. *Energy Storage Materials* **54**, 469-477 (2023).
22. Hong C, Yang G, Wang C. Highly Reversible Zn Electrodeposition Enabled by an Artificial 3D Defect-Rich Conductive Scaffold. *ACS Appl Mater Interfaces* **13**, 54088-54095 (2021).
23. Bayaguud A, Luo X, Fu Y, Zhu C. Cationic Surfactant-Type Electrolyte Additive Enables Three-Dimensional Dendrite-Free Zinc Anode for Stable Zinc-Ion Batteries. *ACS Energy Letters* **5**, 3012-3020 (2020).
24. An Y, Tian Y, Liu C, Xiong S, Feng J, Qian Y. Rational Design of Sulfur-Doped Three-Dimensional $Ti_3C_2T_x$ MXene/ZnS Heterostructure as Multifunctional Protective Layer for Dendrite-Free Zinc-Ion Batteries. *ACS Nano* **15**, 15259-15273 (2021).
25. Cao Q*, et al.* Regulating Dendrite-Free Zinc Deposition by 3D Zincopilic Nitrogen-Doped Vertical Graphene for High-Performance Flexible Zn-Ion Batteries. *Advanced Functional Materials* **31**, (2021).
26. Park JB, Choi C, Park JH, Yu S, Kim DW. Synergistic Design of Multifunctional Interfacial Zn Host toward Practical Zn Metal Batteries. *Advanced Energy Materials* **12**, (2022).
