# Supplementary material for: 3D hierarchical graphene matrices enable stable Zn anodes for aqueous Zn batteries
Source: Nat Commun. 2023 Jul 14;14:4205. doi: 10.1038/s41467-023-39947-8 (PMC10349079; doi:10.1038/s41467-023-39947-8)
Supplement: Supplementary file 3 — Description of Additional Supplementary Files [file 41467_2023_39947_MOESM3_ESM.pdf]

Description of Additional Supplementary Files

File Name: Supplementary Movie 1
Description: The ion concentration distribution of the 3D-RFGC matrices from the top views.

File Name: Supplementary Movie 2
Description: The ion concentration distribution of the 3D-RC matrices along the vertical direction.

File Name: Supplementary Movie 3
Description: The ion concentration distribution of the 3D-LC matrices along the vertical direction.

File Name: Supplementary Movie 4
Description: The ion concentration distribution of the 3D-LFGC matrices along the vertical direction.

File Name: Supplementary Movie 5
Description: The local current density distribution of the 3D-RC matrices along the vertical direction.

File Name: Supplementary Movie 6
Description: The local current density distribution of the 3D-LFGC matrices along the vertical direction.
