# Supplementary figures and images for: 3D hierarchical graphene matrices enable stable Zn anodes for aqueous Zn batteries
Source: Nat Commun. 2023 Jul 14;14:4205. doi: 10.1038/s41467-023-39947-8 (PMC10349079; doi:10.1038/s41467-023-39947-8)

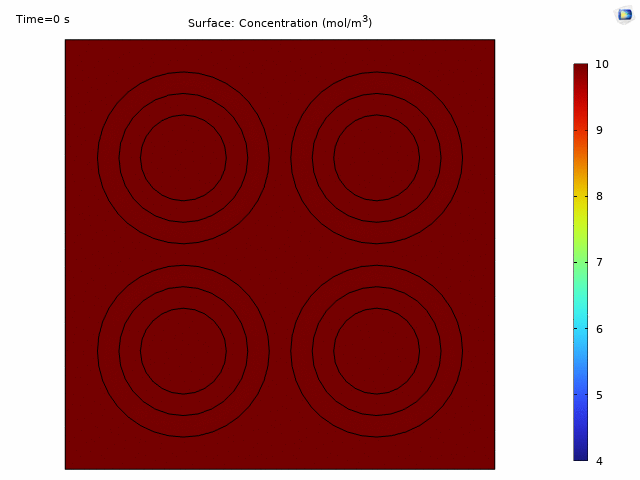

Supplement: Supplementary file 4 — Supplementary Movie 1 [file 41467_2023_39947_MOESM4_ESM.gif]

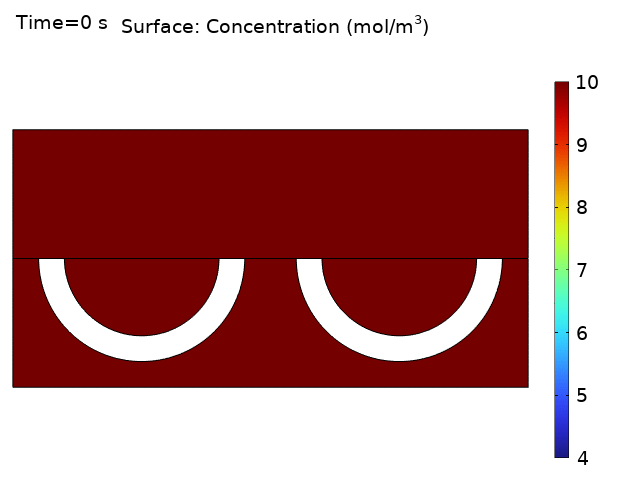

Supplement: Supplementary file 5 — Supplementary Movie 2 [file 41467_2023_39947_MOESM5_ESM.gif]

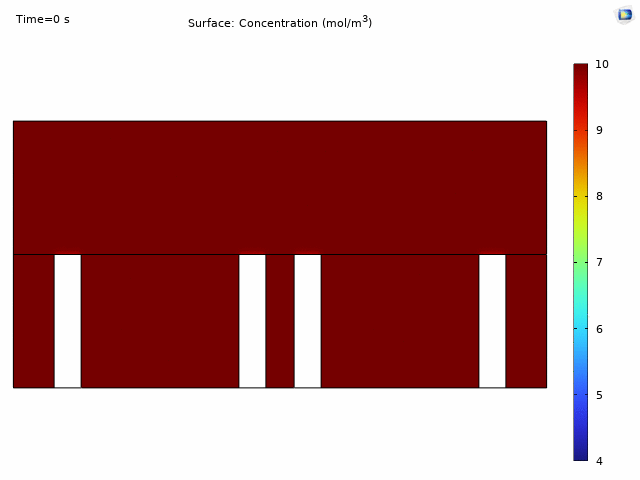

Supplement: Supplementary file 6 — Supplementary Movie 3 [file 41467_2023_39947_MOESM6_ESM.gif]

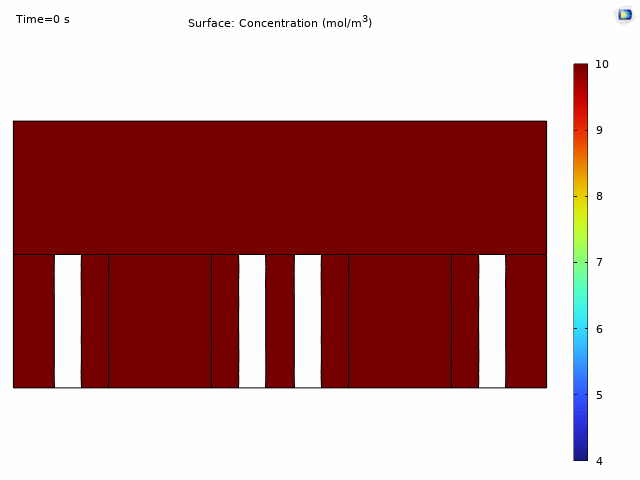

Supplement: Supplementary file 7 — Supplementary Movie 4 [file 41467_2023_39947_MOESM7_ESM.gif]

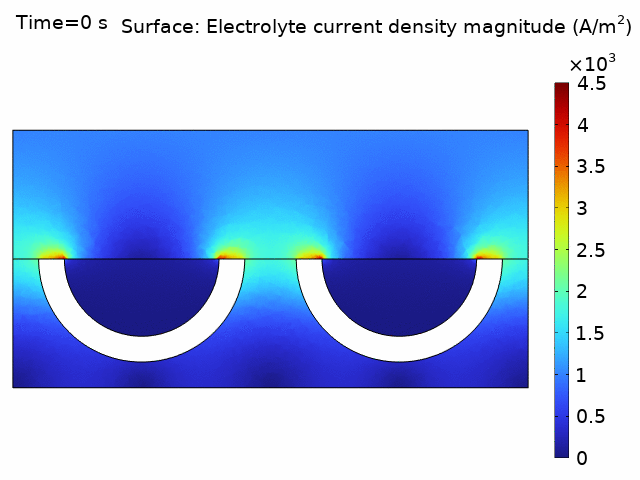

Supplement: Supplementary file 8 — Supplementary Movie 5 [file 41467_2023_39947_MOESM8_ESM.gif]

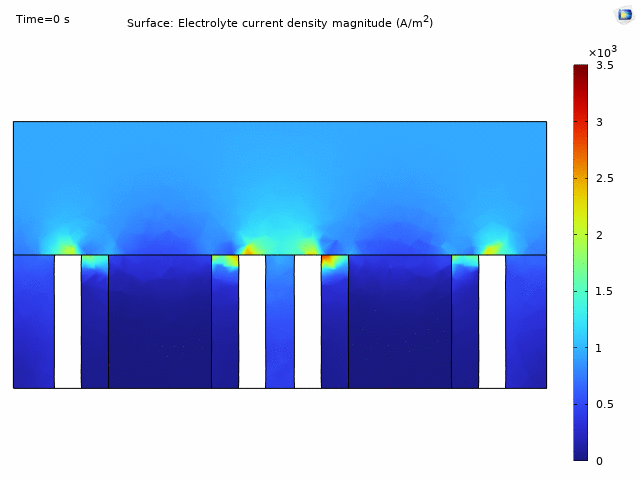

Supplement: Supplementary file 9 — Supplementary Movie 6 [file 41467_2023_39947_MOESM9_ESM.gif]

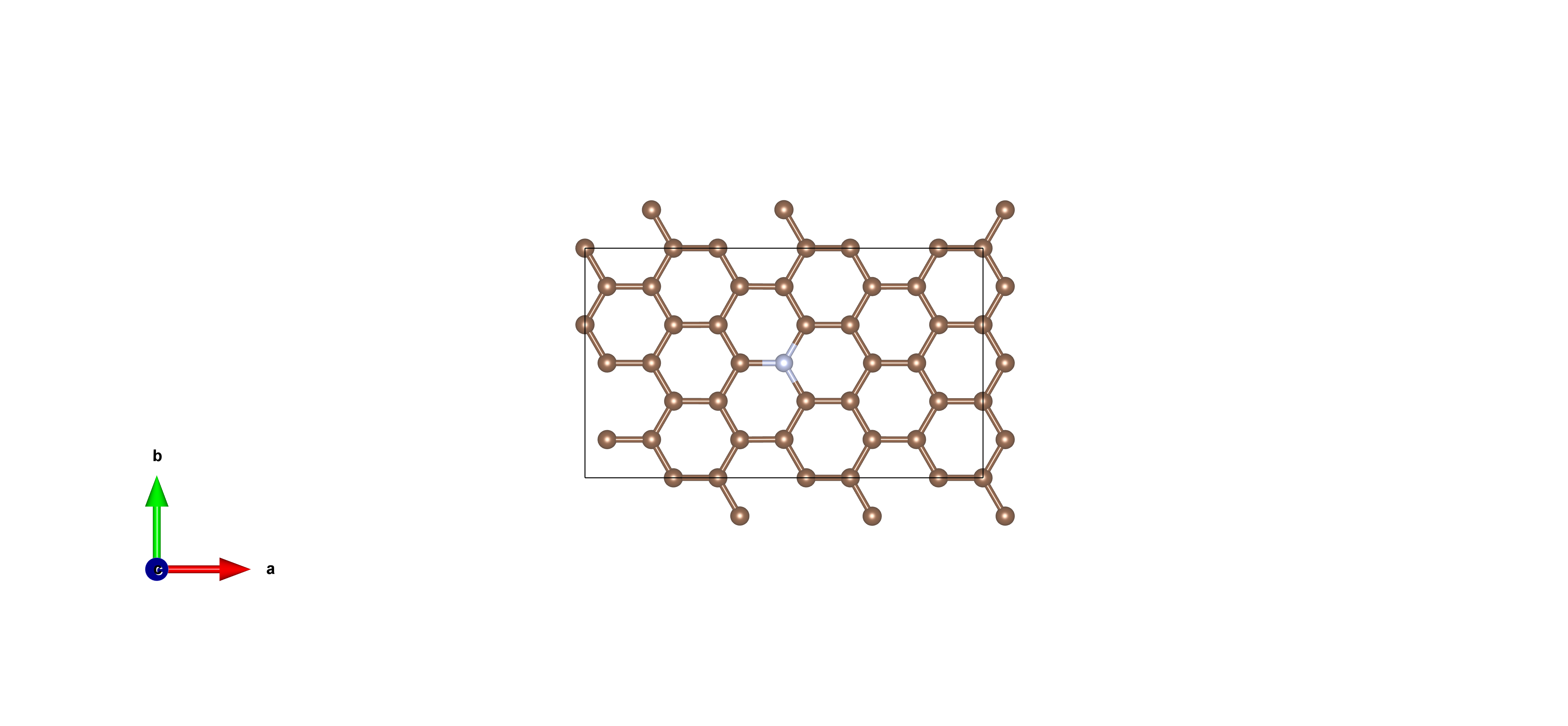

Supplement: Supplementary file 11 — Source Data [file 41467_2023_39947_MOESM11_ESM.zip › RAW DATA/Supplementary information/Supplementary Fig.13 and 14/cif/Nitrogen-Doped Graphene/CONTCAR-C-N.tif]

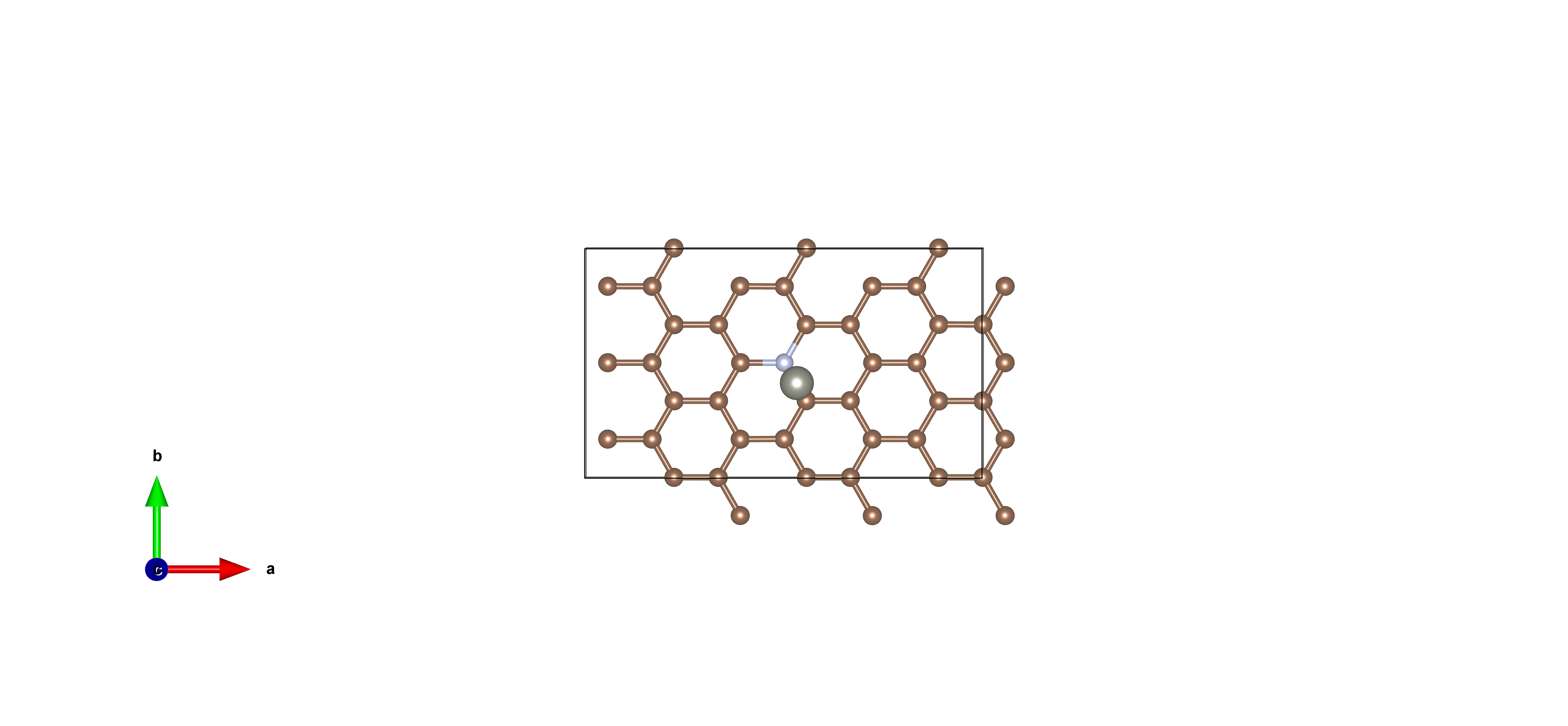

Supplement: Supplementary file 11 — Source Data [file 41467_2023_39947_MOESM11_ESM.zip › RAW DATA/Supplementary information/Supplementary Fig.13 and 14/cif/Nitrogen-Doped Graphene/CONTCAR-bridge.tif]

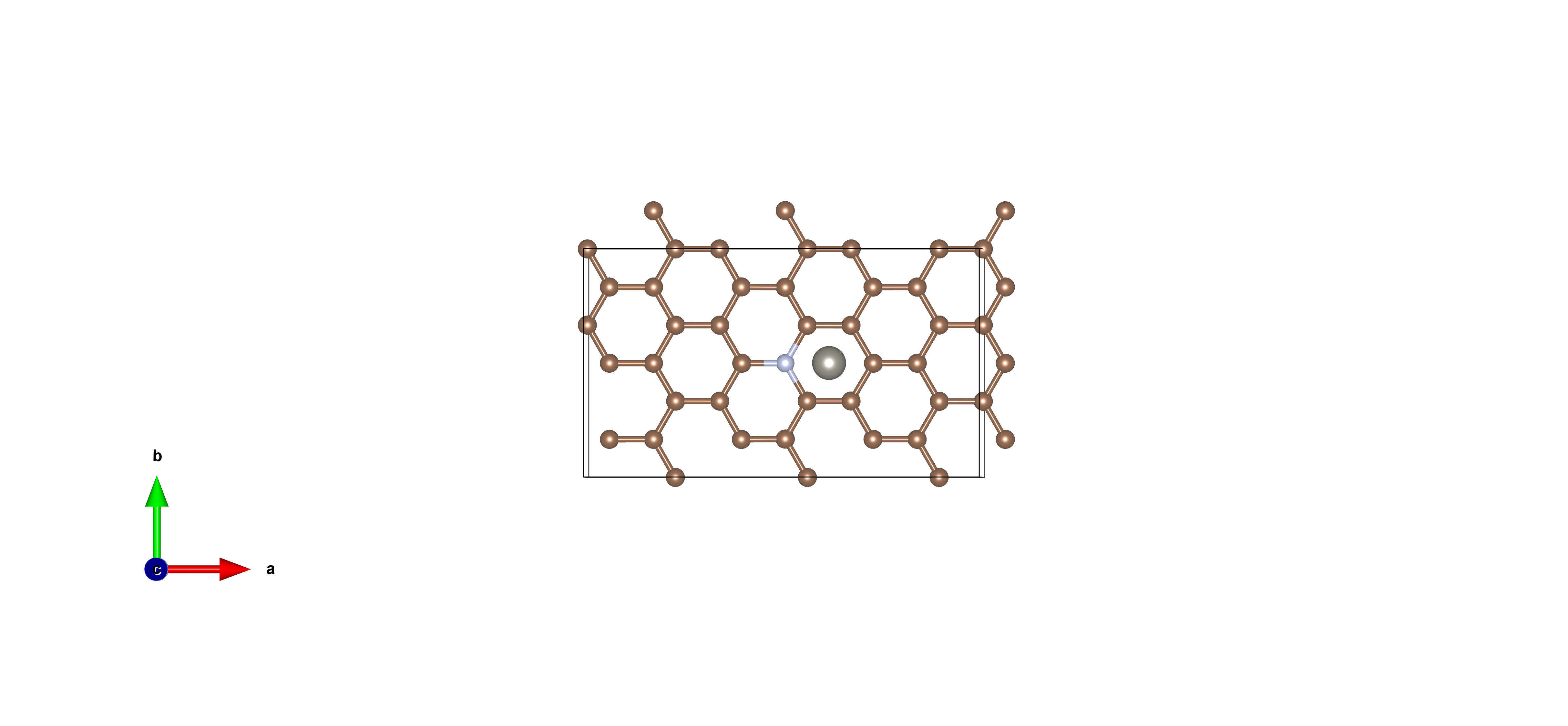

Supplement: Supplementary file 11 — Source Data [file 41467_2023_39947_MOESM11_ESM.zip › RAW DATA/Supplementary information/Supplementary Fig.13 and 14/cif/Nitrogen-Doped Graphene/CONTCAR-hollow.tif]

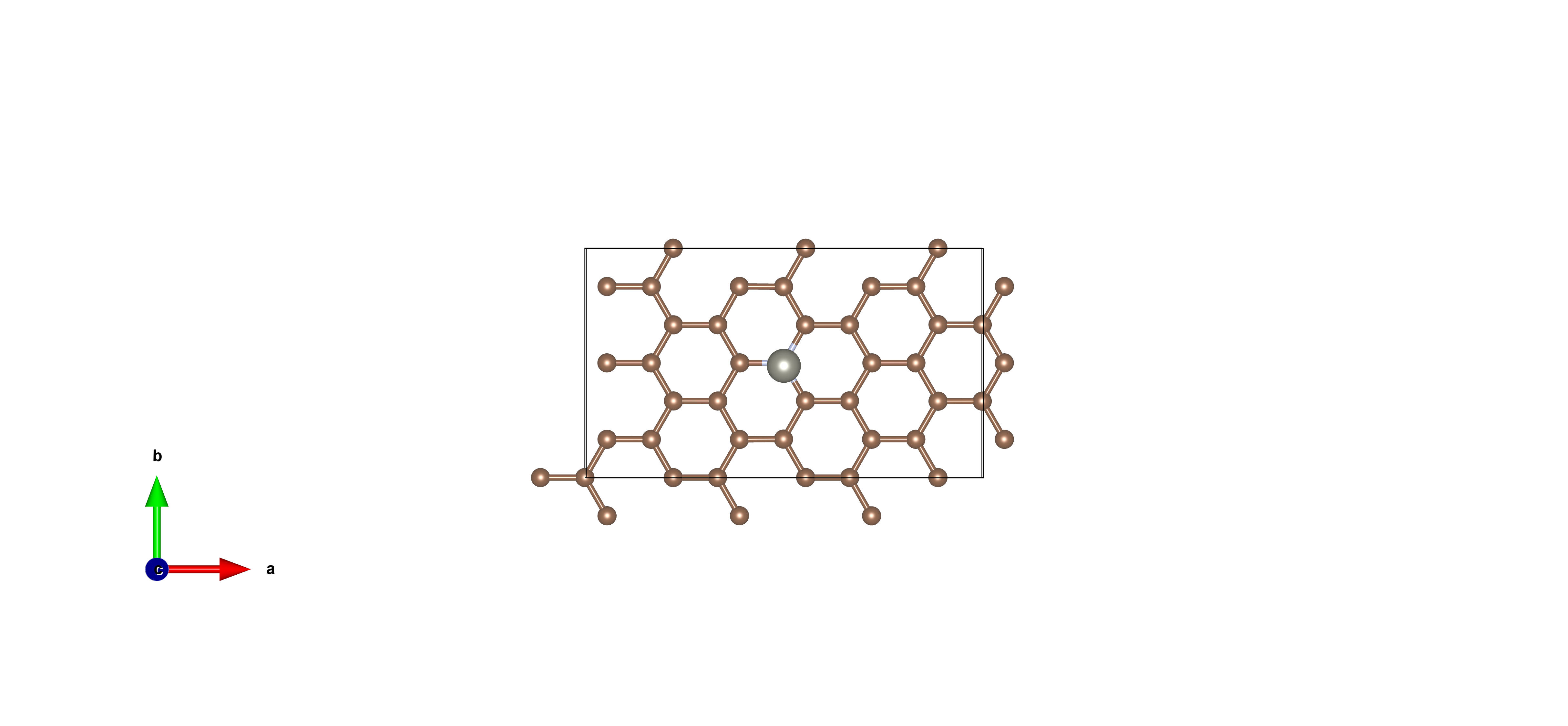

Supplement: Supplementary file 11 — Source Data [file 41467_2023_39947_MOESM11_ESM.zip › RAW DATA/Supplementary information/Supplementary Fig.13 and 14/cif/Nitrogen-Doped Graphene/CONTCAR-top.tif]
